# Comparative genomics applied to *Mucor* species with different lifestyles

**DOI:** 10.1186/s12864-019-6256-2

**Published:** 2020-02-10

**Authors:** Annie Lebreton, Erwan Corre, Jean-Luc Jany, Loraine Brillet-Guéguen, Carlos Pèrez-Arques, Victoriano Garre, Misharl Monsoor, Robert Debuchy, Christophe Le Meur, Emmanuel Coton, Georges Barbier, Laurence Meslet-Cladière

**Affiliations:** 1 Univ Brest, Laboratoire Universitaire de Biodiversité et Ecologie Microbienne, F-29280 Plouzané, France; 2 Station Biologique de Roscoff, Plateforme ABiMS, CNRS: FR2424, Sorbonne Université (UPMC), Paris VI, Place Georges Teissier, 74 29682 Roscoff Cedex, BP France; 3 CNRS, Integrative Biology of Marine Models (LBI2M), Station Biologique de Roscoff (SBR), Sorbonne Université, 29680 Roscoff, France; 4 Department of Genetics and Microbiology, Faculty of Biology, University of Murcia, 30100 Murcia, Spain; 5 Institute for Integrative Biology of the Cell (I2BC), CEA, CNRS, University Paris-Sud, Université Paris-Saclay, CEDEX 91198 Gif-sur-Yvette, France

**Keywords:** Early divergent fungi, Genome, Adaptation, CAZymes, Peptidases, Iron uptake

## Abstract

**Background:**

Despite a growing number of investigations on early diverging fungi, the corresponding lineages have not been as extensively characterized as *Ascomycota* or *Basidiomycota* ones. The *Mucor* genus, pertaining to one of these lineages is not an exception. To this date, a restricted number of *Mucor* annotated genomes is publicly available and mainly correspond to the reference species, *Mucor circinelloides,* and to medically relevant species. However, the *Mucor* genus is composed of a large number of ubiquitous species as well as few species that have been reported to specifically occur in certain habitats. The present study aimed to expand the range of *Mucor* genomes available and identify potential genomic imprints of adaptation to different environments and lifestyles in the *Mucor* genus.

**Results:**

In this study, we report four newly sequenced genomes of *Mucor* isolates collected from non-clinical environments pertaining to species with contrasted lifestyles, namely *Mucor fuscus* and *Mucor lanceolatus*, two species used in cheese production (during ripening), *Mucor racemosus*, a recurrent cheese spoiler sometimes described as an opportunistic animal and human pathogen, and *Mucor endophyticus*, a plant endophyte. Comparison of these new genomes with those previously available for six *Mucor* and two *Rhizopus* (formerly identified as *M. racemosus*) isolates allowed global structural and functional description such as their TE content, core and species-specific genes and specialized genes. We proposed gene candidates involved in iron metabolism; some of these genes being known to be involved in pathogenicity; and described patterns such as a reduced number of CAZymes in the species used for cheese ripening as well as in the endophytic isolate that might be related to adaptation to different environments and lifestyles within the *Mucor* genus.

**Conclusions:**

This study extended the descriptive data set for *Mucor* genomes, pointed out the complexity of obtaining a robust phylogeny even with multiple genes families and allowed identifying contrasting potentially lifestyle-associated gene repertoires. The obtained data will allow investigating further the link between genetic and its biological data, especially in terms of adaptation to a given habitat.

## Background

The *Mucor* genus belongs to the most prominent order of the *Mucorales*, a phylogenetically ancient group of fungi pertaining to the “early diverging fungi” [1]. From the first microscopic observation of a *Mucor* specimen in 1665 up until now, several hundreds of potential *Mucor* species have been reported [2]. *Mucor* species are common and predominantly saprotrophs [3]. These ubiquitous microorganisms may colonize multiple and contrasted environments from dungs or dead plant materials to plant and animal tissues. Members of the *Mucor* genus have an ambivalent impact on human activities. Regarding their negative impact, some *Mucor* species, in particular the thermotolerant species *Mucor indicus, Mucor ramosissimus* and seven members of the *Mucor circinelloides* complex [4] have been shown to be human and animal pathogens responsible for mucormycosis [2]. Mucormycosis has been recently described as the third most common angioinvasive fungal infection and can lead to death [5]. Another negative impact concerns the ability of different species of the genus to spoil raw and transformed foods and feeds [6]. On the contrary, some *Mucor* species have an important biotechnological potential thanks to their high growth rates in a large range of temperatures [7], existence of a yeast state in certain *Mucor* spp. [8], and high proteolytic and lipolytic enzymatic activities [9], making them good candidates for biotechnology. Interestingly, some species are also used in food manufacturing of Asian fermented food production (such as ragi, tempeh, furu or mureha) or for French cheese ripening (such as Tomme or Saint-Nectaire) [2].

The increasing number of infections associated with *Mucor* species, as well as the biotechnological potential of the genus, have led to a large effort to better know these fungi. In this context, Vongsangnak et al. proposed a metabolic network of the oleaginous strain *Mucor circicinelloides* WJ11 [10, 11], Corrochano et al. shed new light on *Mucor* sensory perception [12], and multiple genes potentially involved in virulence were investigated and discovered in *Mucor* spp. [13–21]. Following the annotation of the first *Mucor* genome sequence (*Mucor circinelloides* CBS 277.49), researches on *Mucor* benefited and will continue to benefit from different sequencing projects including the Zygolife initiative (http://zygolife.org/home/) which aims to provide a better phylogenetical classification to the formerly called Zygomycetes which include the *Mucor* genus (see [1]).

This phylogenetical classification appears to be challenging as stated by inconsistencies among previous works; e.g. *Mucor indicus* CDC-B7402 placement was modified between the phylogeny of Álvarez et al. and that of Whalter et al., *Mucor endophyticus* CBS 385–95 placement was modified between Whalter et al. and Lebreton et al. respective studies [22–24]. Moreover, the uncertain taxonomic assignment of some *Mucor* isolates used in published studies may lead to confusion. For example, following genomic studies, the 97–1192 isolate was reassigned from *Mucor racemosus* to *Rhizopus oryzae* and isolate CDC-B9738 (initially *Rhizopus microsporus)* was consecutively assigned to *Mucor racemosus* by Chibucos et al. and more recently reassigned to *R. microsporus* by Gryganskyi et al. [13, 25]*.* As stated by Gryganskyi et al., the closely related genus *Rhizopus* cannot be deciphered with a single or even a handful of gene families [25]. However, the range of *Mucor* genome sequences exploited is limited and those available with annotations even scarcer. Furthermore, these genome sequencing projects are mainly limited to *Mucor* species with a biotechnological or pathogenic potential. Indeed, at the beginning of this study, only six *Mucor* annotated genomes were freely available, five of them corresponding to isolates pertaining to the *M. circinelloides* complex.

The present study aimed to use comparative genomics to identify potential genomic imprints of adaptation to different environments and lifestyles in the *Mucor* genus. To do so, four genomes, corresponding to *Mucor fuscus* UBOCC-A-109160 and *Mucor lanceolatus* UBOCC-A-109153 (used in cheese production, during ripening), *Mucor racemosus* UBOCC-A-109155 (a cheese spoiler sometimes described as an opportunistic animal and human pathogen) [26] and *Mucor endophyticus* CBS 385–95 (a plant endophyte [27]), were sequenced, annotated and compared to those of six publicly available *Mucor* and two *Rhizopus* (formerly identified as *Mucor*) isolates.

## Results

### Genome description

#### Genome sequences and assembly

The genomes of *M. fuscus* UBOCC-A-109160, *M. lanceolatus* UBOCC-A-109153, *M. racemosus* UBOCC-A-109155 and *M. endophyticus* CBS 385–95 were sequenced, assembled and annotated in the context of this study. Their genome features were compared to eight previously sequenced and annotated genomes from *Mucor* isolates (*n* = 6) and *Rhizopus* isolates formerly identified as *Mucor* spp. (*n* = 2) [12, 13, 28] (Table 1). Due to the recent reassignment of the *Mucor circinelloides* complex members to distinct species [4], isolates identified as *M. circinelloides* were renamed according to their current phylogenetic placement: *Mucor circinelloides* CBS 277.49 was renamed into *Mucor lusitanicus* CBS 277.49, *Mucor circinelloides* CDC-B5328 into *Mucor velutinosus* CDC-B5328 and *Mucor circinelloides* NBRC 6742 (synonym of *Mucor ambiguus* NBRC 6742) into *Mucor griseocyanus* NBRC 6742. For simplicity, with the exception of *M. circinelloides* and *R. microsporus* representatives (two isolates were analysed both for *M. circinelloides* and *R. microsporus* species), the different isolates investigated will, hereafter, be named by their species name.

**Table 1:**
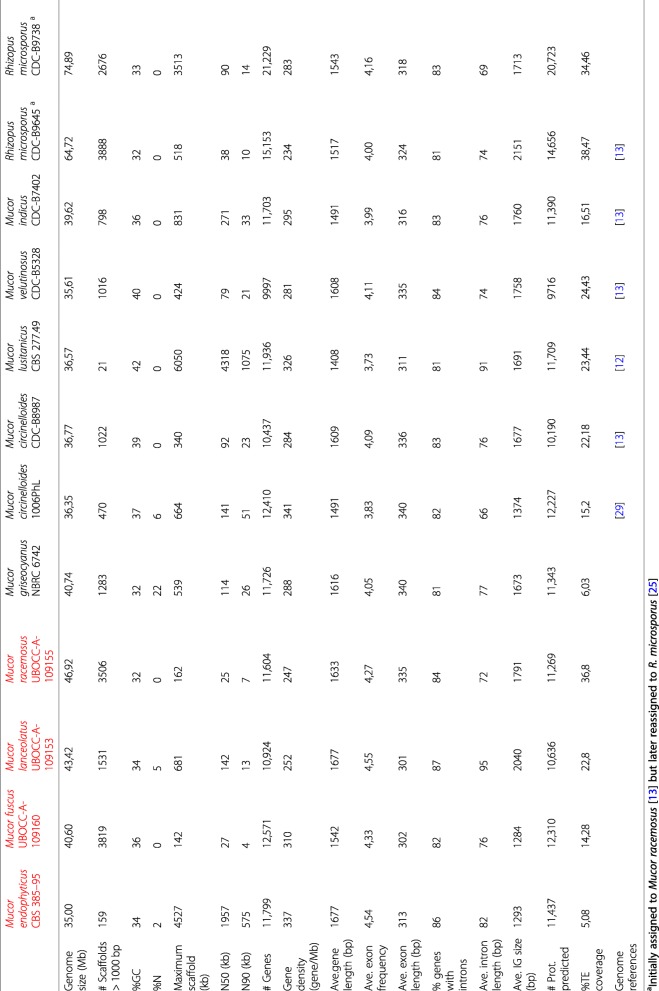
Genome assembly features and structural annotation of the studied Mucor and Rhizopus isolates. The four isolates sequenced in the context of this work are in red. IG: intergenic regions, TE: transposable elements

The number of scaffolds in *M. lusitanicus* assembly was the lowest with 21 scaffolds. *M. endophyticus* was the second less fragmented assembly with 159 scaffolds. The 10 other assemblies were composed of 470 to 3888 scaffolds (Table 1).

Despite these differences in genome fragmentation, at least 95% of the 290 single copy fungal orthologous genes searched by BUSCO were found complete in all genomes. It is worth noting that respectively 52 and 87% of the searched genes were found duplicated in *Rhizopus microsporus* CDC-B9645 and *Rhizopus microsporus* CDC-B9738 genomes whereas, in the other genomes, duplicated genes only represented 17 to 26% of the searched genes. The two *Rhizopus microsporus* isolates also exhibited the highest genome size with 65 Mb and 75 Mb for CDC-B9645 and CDC-B9738 isolates, respectively, while the average size for the other geomes is approximately 39 Mb. The endophytic species *M. endophyticus*, had the smallest genome size (35 Mb) (Table 1).

#### Genome annotation

The number of predicted genes was in accordance with genome sizes. In *Mucor* species, the number of predicted genes fluctuated from 9997 to 12,571, i.e. a gene density ranging from 247 to 341 genes/Mb, while 15,153 and 21,229 genes were predicted for *R. microsporus* CDC-B9645 and CDC-B9738, i.e. a gene density of 234 and 283 genes/Mb, respectively (Table 1).

Gene characteristics were well conserved among the *Mucor* and *Rhizopus* genomes: the average gene length was 1568 bp, 83% of genes had predicted introns, genes had an average frequency of 4.1 exons and average intron size was approximately 60 bp.

**Fig. 1:**
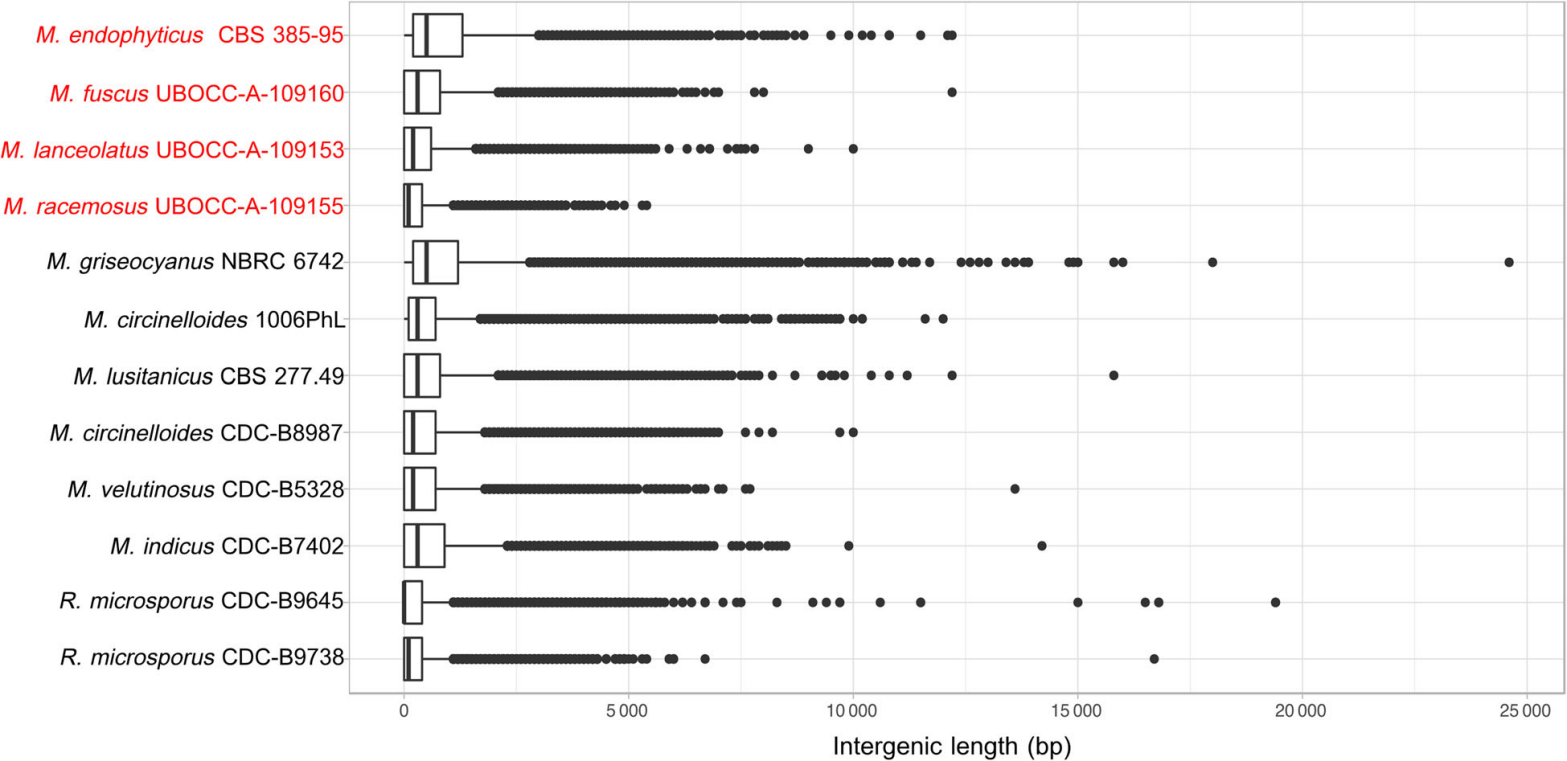
Repartition of the intergenic length within the 12 studied *Mucor* and *Rhizopus* genomes. The four isolates sequenced in the context of this study are in red. The boxes represent intergenic length harbored by 25 to 75% (sorted by length) of the intergenic regions, the line within the box represent the median length, dots represent each value corresponding to the 10% longest intergenic units represented

Noteworthy, within all genomes, the intergenic distance was variable: 25% of intergenic regions were shorter than ~ 300 bp whereas the largest intergenic regions exceeded 20 kb. When repeated elements were taken into account for the analysis, regions up to 15 kb with neither gene nor repeated elements were still detected (Fig. 1).

### Genome comparisons

#### Phylogenomic reconstruction

**Fig. 2:**
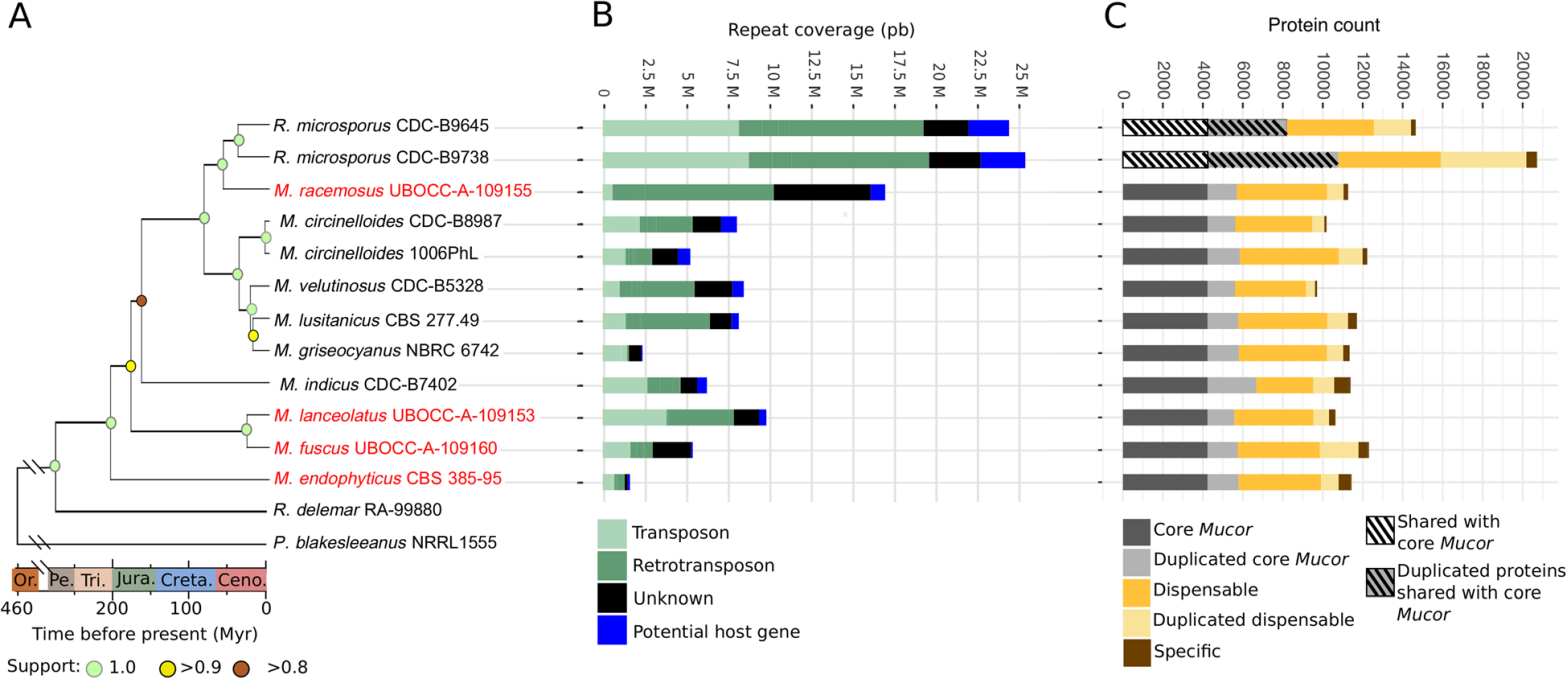
Representation of the phylogenomic tree, repetitions coverage and gene number for the genomes pertaining to the studied *Mucor* and *Rhizopus* isolates (initially assigned to *M. racemosus*). **a** Phylogenomic tree of the studied isolates reconstructed with RAxML based on 51 single copy ortholog families using *R. delemar* RA-99880 and *P. blakesleeanus* NRRL1555 as outgroup and calibrated on time scale with r8S program. The branch length corresponds to the estimated time before present. Color dots represent bootstrap support. The four isolates sequenced in this study are in red. **b** Representation of the repetitions coverage in each genome. Unknown: potential transposable element that could not be identified. **c** Representation of the protein numbers in the studied isolates. Duplicated proteins count represent extra copy number of each gene family

The 181,601 proteins predicted from the 10 *Mucor*, three *Rhizopus* and the *Phycomyces blakesleeanus* genomes were grouped in 20,588 orthogroups. Among them, 4240 orthogroups were composed of predicted proteins belonging to all genomes while 64 were composed of single copy orthologs. Among the 64-single copy orthogroups, 51 were selected to reconstruct the species tree using Maximum Likelihood and Bayesian methods, *R. delemar* and *P. blakesleeanus* being used as outgroups (Fig. 2a). The present study confirmed the recently described phylogenetic positions of the former *M. circinelloides* formae: *M. griseocyanus*, *M. lusitanicus* and *M. velutinosus* as distinct clades from *M. circinelloides* [4]. The placement of *M. indicus* was altered compared to the topology obtained by Whalter et al. but concurred with the topology published by Álvarez et al. [22, 24]. Similarly, the placement of *R. microsporus* CDC-B9645 and *R. microsporus* CDC-B9738 was concordant with the topology of Chibucos et al. which identified these two isolates as *M. racemosus*, but clearly differentiated from the one by Gryganskyi et al. in which the two isolates were clustered with *R. microsporus* isolates (and renamed accordingly) [13, 25]. This phylogenetic position was not affected when four previously sequenced *R. microsporus* were added to the phylogenomic reconstruction (Additional file 1: Figure S1). These results clearly raise questions concerning their actual position and the genetic bases associated with these incongruences. The other studied isolates had concordant phylogenetic placements with previously published studies [13, 22, 23, 25].

From the biological point of view, *M. fuscus* and *M. lanceolatus* that are close in the tree, have mainly been encountered in cheeses, the basal singleton *M. endophyticus* has only been described as a wheat leaf endophyte, whereas the other *Mucor* species, grouped in the same clade, are usually described as potential human pathogenic species.

#### Transposable elements

The studied genomes contained contrasting transposable element (TE) coverages (Fig. 2b). The plant endophyte, *M. endophyticus*, held the lowest TE coverage (5%) whereas the ubiquitous *M. racemosus* contained the highest TE coverage (37%). Even between close species, TE coverage and composition differed notably, e.g. between *M. lanceolatus* and *M. fuscus* or between *M. griseocyanus* and the other species from the *M. circinelloides* complex (*M. circinelloides*, *M. lusitanicus* and *M. velutinosus*). *M. lanceolatus* showed higher TE coverage than *M. fuscus* (23 and 14%, respectively) for both transposons and retrotransposons but the predicted elements in *M. lanceolatus* were mainly non-autonomous (i.e. not including all the domains necessary for their transposition). On the contrary, almost all *M. fuscus* predicted TE were autonomous, the main represented category being terminal inverted repeat (TIR) but few retrotransposons, long interspersed nuclear elements (LINE) and long terminal repeat (LTR), were also detected (Additional file 2: Figure S2).

Among members of the *M. circinelloides* complex, three species displayed a similar pattern in terms of TE composition and, except for one of the *M. circinelloides* isolates (1006PhL)*,* in terms of coverage. The fourth species, *M. griseocyanus,* displayed striking differences from the other species. Indeed, the TE coverage of *M. griseocyanus* was reduced by at least twofold compared to the other members (Fig. 2b). Furthermore, the main TE category was identified as belonging to the helitron order, a marginal category in the three other members of the clade.

#### Predicted proteins repartition

The predicted protein repertoires encoded by the 14 *Mucoromycota* investigated were compared with one another. This led to the identification of a set of core predicted proteins shared by all studied *Mucor* species and to the determination of the proteins also occurring in the studied *R. microsporus* isolates. Dispensable proteins (shared by at least two species) and species-specific proteins (only found in a single species) were also identified (Fig. 2c). For each category, duplicated proteins were identified. Despite the phylogenetic divergence between species, at least one third of the predicted proteins for each species were part of the core predicted proteome and the number of species-specific proteins was restricted.

As suggested by BUSCO results, most of *R. microsporus* predicted proteins were duplicated (Fig. 2c). This result explained the relatively low number of single copy core proteins gathered for phylogeny reconstruction despite a core *Mucor* proteome of approximately 6000 proteins.

#### Evolution of gene families across phylogeny

When using the whole genome dataset, CAFE-, DupliPhyML- and Notung-based analyses yielded non-concordant results (data not shown) with inconsistent placements of expansion/contraction events within the cluster encompassing *R. microsporus* CDC-B9738*, R. microsporus* CDC-B9645 and *M. racemosus*. This behaviour was interpreted as a side effect of a putative whole genome duplication or hybridization compatible with the observations done on the two *R. microsporus* genomes. The phylogenetic placement of these two isolates within the genus *Mucor* being also questioned, we decided to remove them from the CAFE analysis. CAFE identified 44 rapidly evolving gene families on the *M. lanceolatus*/*M. fuscus* branch (pertaining to the two species associated to cheese ripening). Among these families, two were associated to secondary metabolism, namely an acyl-CoA synthetase and a cytochrome p450 encoding gene families, both with reduced number of genes in cheese ripening species (*M. fuscus* and *M. lanceolatus*) compared to other species. A cysteine hydrolase gene family was also reduced in the two cheese-associated species. Another family (less conserved) with genes identified as encoding putative transcriptional activators of glycolytic enzyme was expanded in the latter species genomes. Other families were either unknown or similar to TE sequences.

In the *M. endophyticus* endophyte, at the node separating cheese-associated species from pathogenic species and at the node separating *M. indicus* from other species, 49, 4 and 9 gene families were considered as rapidly evolving, respectively. However, these gene families were either of unknown function or similar to TE sequences.

#### Genes coding for CAZymes, peptidases and small secreted proteins

As different CAZyme and peptidase repertoires are associated to different lifestyles, comparison of the annotations of CAZyme and peptidase genes annotation of *M. lusitanicus* were publicly available in the MycoCosm database (https://mycocosm.jgi.doe.gov/mycocosm/home). However, for the sake of the comparison, those annotations were performed again in this study following the same pipeline as the other studied genomes. This allowed to show that, for *M. lusitanicus*, the number of peptidase genes was found higher (351 against 304) and the number of CAZymes lower (229 against 339) in this study compared to annotation performed by the Join Genome Institute and displayed in the MycoCosm database. Taking this difference into account, the total numbers of CAZyme (155–306) (Table 2) and peptidase (332–404) encoding genes (Table 3) were found concordant with what is observed in other sequenced species of the *Mucoromycota* phylum as indicated in the MycoCosm protein database. In particular, the total number of CAZyme encoding genes in the *Mucor* genomes analysed in this study was lower than what is observed in average in *Ascomycota* and *Basidiomycota* (Additional file 3: Figure S3). Moreover, among CAZyme genes, those encoding glycosyl-transferases (GT) were found more numerous than those encoding glycoside hydrolases (GH) which is usual in the *Mucoromycota* but not in *Dikarya* (Additional file 3: Figure S3).

**Table 2:** Number of genes encoding Carbohydrate Active enZymes (CAZymes). Major CAZymes classes are shown separately, Auxiliary redox enzymes (AA), Carbohydrate-Binding Modules (CBM), Carbohydrate Esterases (CE), Glycoside Hydrolases (GH), Glycoside Transferases (GT) and Polysaccharide Lyases (PL). Enzymes substrats are indicated: Cell Wall (CW), Fungal Cell Wall (FCW) and Plant Cell Wall (PCW). The four isolates sequenced in the context of this study are in red

Isolate	AA	CBM	CE	GH	GT	PL	Total CAZymes	CW	FCW	PCW
	12	2	15	56	67	2	155	10	19	8
	12	7	18	68	107	1	214	12	21	12
	10	5	16	62	105	2	201	11	19	12
	11	5	20	81	115	2	235	18	22	14
*M. griseocyanus* NBRC_6742	12	7	25	83	113	2	243	18	25	13
*M. circinelloides* 1006PhL	15	7	23	83	121	2	252	18	24	14
*M. circinelloides* CDC-B8987	15	7	21	83	110	2	239	17	24	14
*M. lusitanicus* CBS 277.49	11	5	23	75	112	2	229	16	22	12
*M. velutinosus* CDC-B5328	16	6	24	78	112	2	239	17	22	13
*M. indicus* CDC-B7402	25	11	34	96	136	3	306	20	28	15

**Table 3:** Number of genes encoding proteolytic enzymes (peptidases) and their inhibitors (MEROPS database). The four isolates newly sequenced in the context of this study are in red

Peptidases							
Isolate	Aspartic	Cysteine	Metallo	Serine	Threonine	Total peptidases	Inhibitors
	31	80	107	118	19	355	11
	28	75	103	117	21	344	10
	24	77	101	115	20	337	10
	31	85	100	117	20	353	12
*M. griseocyanus* NBRC_6742	32	84	101	110	20	347	10
*M. circinelloides* 1006PhL	32	82	101	114	20	349	13
*M. circinelloides* CDC-B8987	30	78	94	109	21	332	11
*M. lusitanicus* CBS 277.49	34	86	103	108	20	351	10
*M. velutinosus* CDC-B5328	29	85	103	109	21	347	13
*M. indicus* CDC-B7402	44	88	114	132	26	404	11

**Fig. 3:**
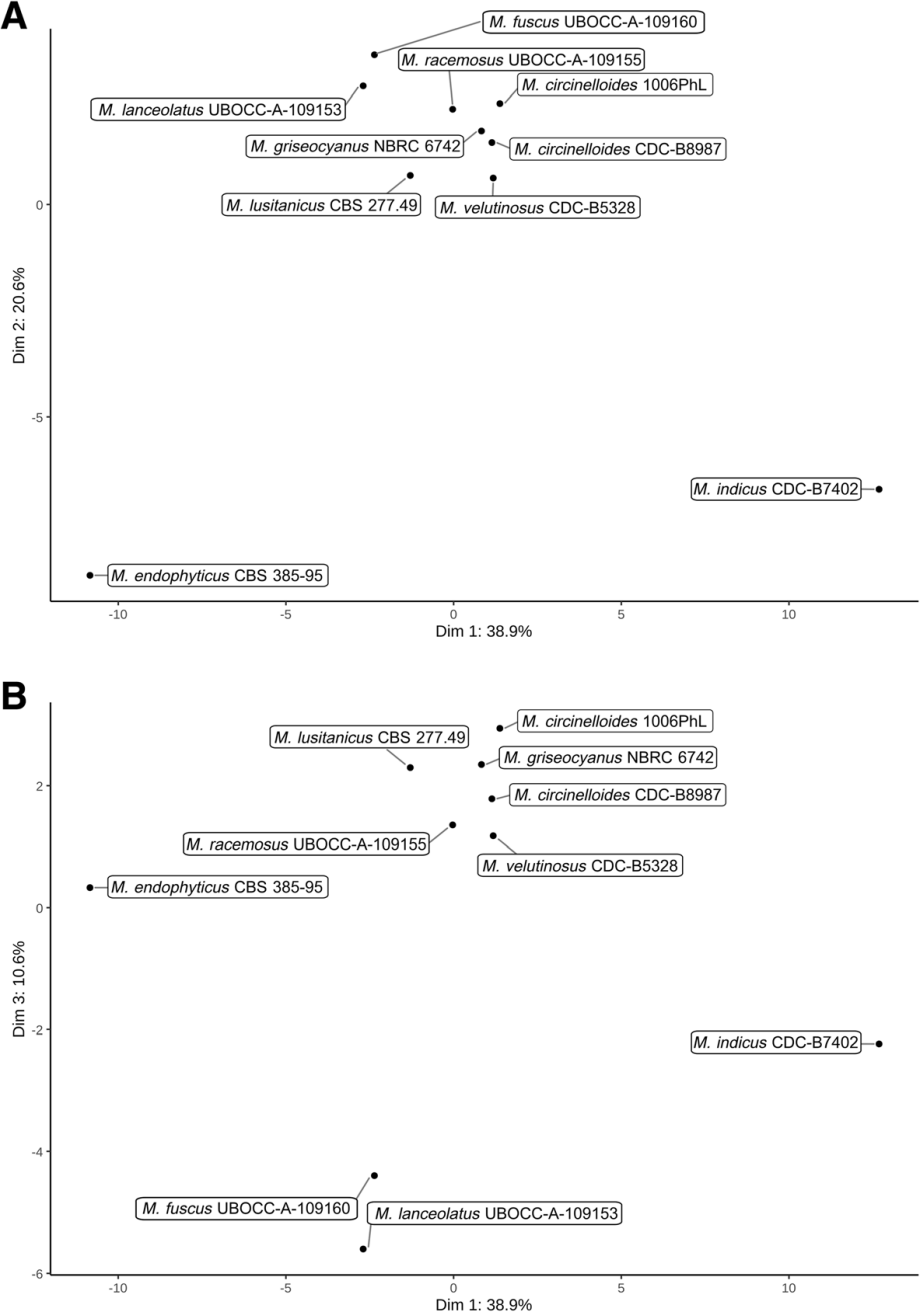
Representation of *Mucor* isolates repartition within the PCA analysis on their CAZYmes family proteins content. **a** Representation of the first two dimensions. **b** Representation of dimensions 1 and 3

Clear differences in terms of CAZyme encoding gene composition among the different genomes were detected. Among the investigated species, *M. indicus* possessed the highest content in all the CAZyme gene classes. In contrast, the number of all classes of CAZyme genes (except those encoding redox enzymes classified in auxiliary activities -AA-) was drastically reduced in *M. endophyticus*. The number of genes coding for catabolic CAZymes was also reduced in *M. fuscus* and *M. lanceolatus*. Interestingly, these reductions were noticeable for genes coding for cell wall degradation enzymes and more specifically in plant cell wall (PCW) degradation enzymes in *M. endophyticus* (Table 2). Differences among groups of species sharing a same putative lifestyle in terms of number of CAZyme families encoding genes were illustrated by the principal component analysis (PCA) of CAZymes in particular along axes 1, 2 and 3 (Fig. 3). The first axis represented the opposition between the thermophilic opportunistic pathogen *M. indicus* and the endophyte *M. endophyticus*. This axis was constructed using mainly 28 CAZyme families (correlation above 0.75) all contracted in *M. endophyticus* compared to *M. indicus*, the other species presenting in almost all cases an intermediary pattern.

The second axis opposed *M. endophyticus* and *M. indicus* to all the other species. Three CAZyme families were mainly involved (correlation above 0.75) in this axis construction: GH8, GH20 and GT77. It is noteworthy that the GH20 β-hexosaminidase family was expanded in *M. endophyticus* and *M. indicus*, whereas the GH8 and GT77 tend to be reduced in the two latter species. The third axis represented the opposition between two species used for cheese ripening (*M. fuscus* and *M. lanceolatus*) and all the other species except *M. indicus*, the latter one presenting an intermediary pattern. Only four CAZymes families were mainly involved (correlation above 0.75) in this axis construction: GH9, GH37 and GT4 were contracted and GH45 expanded in *M. fuscus* and *M. lanceolatus* compared to almost all the other studied species.

Noteworthy, this analysis highlighted the GH3, GH5 (first axis) and GH9 (third axis) CAZyme families which are among the major cellulases involved in plant cell wall degradation. They were less represented in *M. endophyticus* and to a lesser extent in *M. fuscus* and *M. lanceolatus* (Additional file 4: Table S1).

**Fig. 4:**
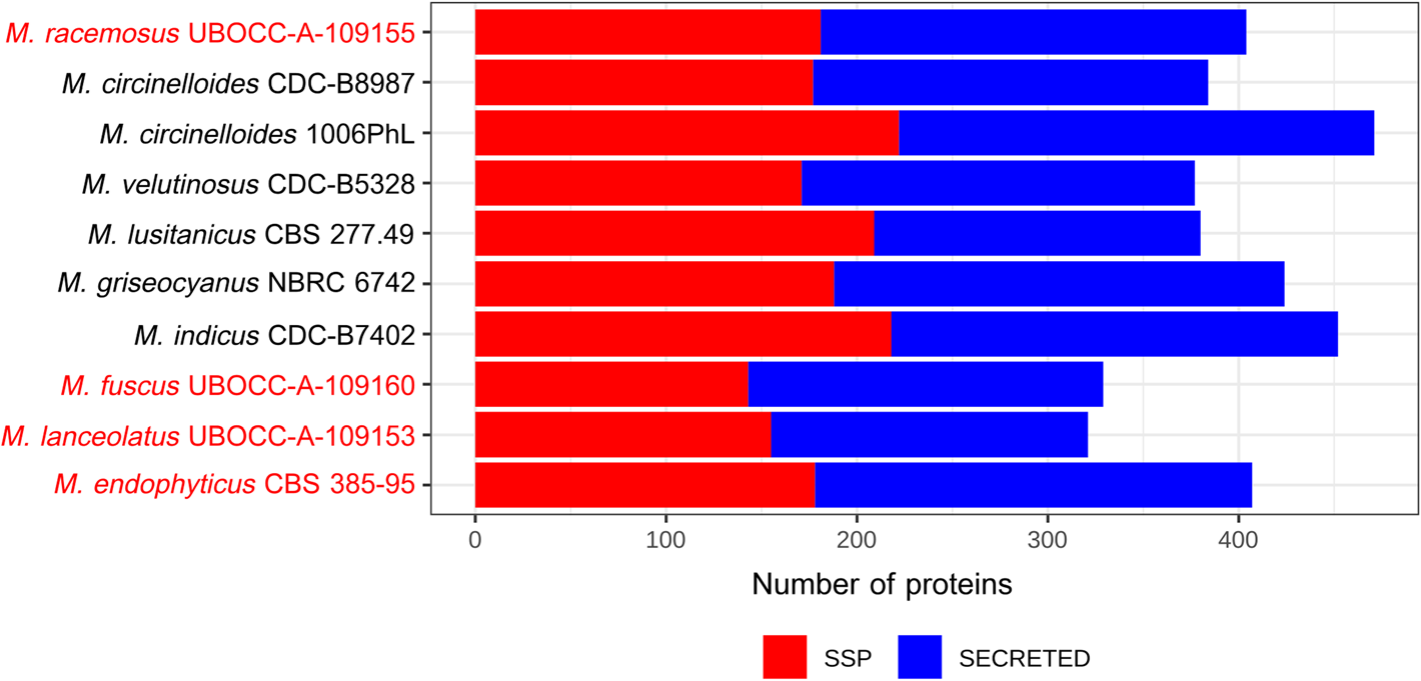
Representation of the number of secreted proteins within the genomes of the studied *Mucor* isolates. SSP: secreted proteins containing less than 300 aa. Secreted: secreted proteins with more than 300 aa. The four isolates sequenced in the context of this study are in red

Noteworthy, in addition to its expanded CAZyme repertoire, *M. indicus* also displayed the largest peptidase (Table 3) repertoire and, with *M. circinelloides* isolate 1006PhL, the highest frequency of genes coding for secreted proteins and small secreted proteins (SSPs) (Fig. 4). In contrast, *M. fuscus* and *M. lanceolatus* exhibited the lowest frequencies of secreted protein and SSP encoding genes (Fig. 4).

#### Genes involved in secondary metabolism

As secondary metabolism genes can be associated with habitat adaptation, genes encoding PKS, NRPS, TPS and DMATS were investigated. Among genes associated with terpene biosynthesis, some corresponding to squalene cyclases, squalene synthases, bifunctional lycopene cyclases, squalene/phytoene synthases and geranylgeranyl pyrophosphate (*ggpp*) synthases were identified in each species. The number of genes in each category was well conserved among the different species (Table 4).

**Table 4:**
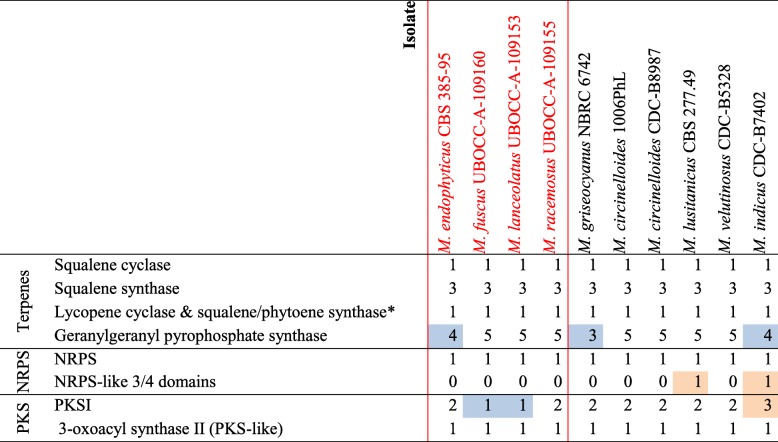
Number of genes involved in secondary metabolites found in the 10 studied *Mucor* isolates. The four species sequenced in this study are in red. For each gene category, maxima are highlighted in orange and minima in blue

^*^These genes encode a putative bifunctional enzyme

In all studied species, a single complete *nrps* gene (i.e. having at least one condensation domain, one carrier domain, one phosphopantetheine attachment site and 1 AMP-binding domain) was detected. Three other genes containing three of the four mandatory NRPS domains were found in *M. indicus*, both of them lacking the condensation domain, and one in *M. lusitanicus*, lacking the AMP-binding domain. In all species, no gene coding for DMATS were identified.

**Fig. 5:**
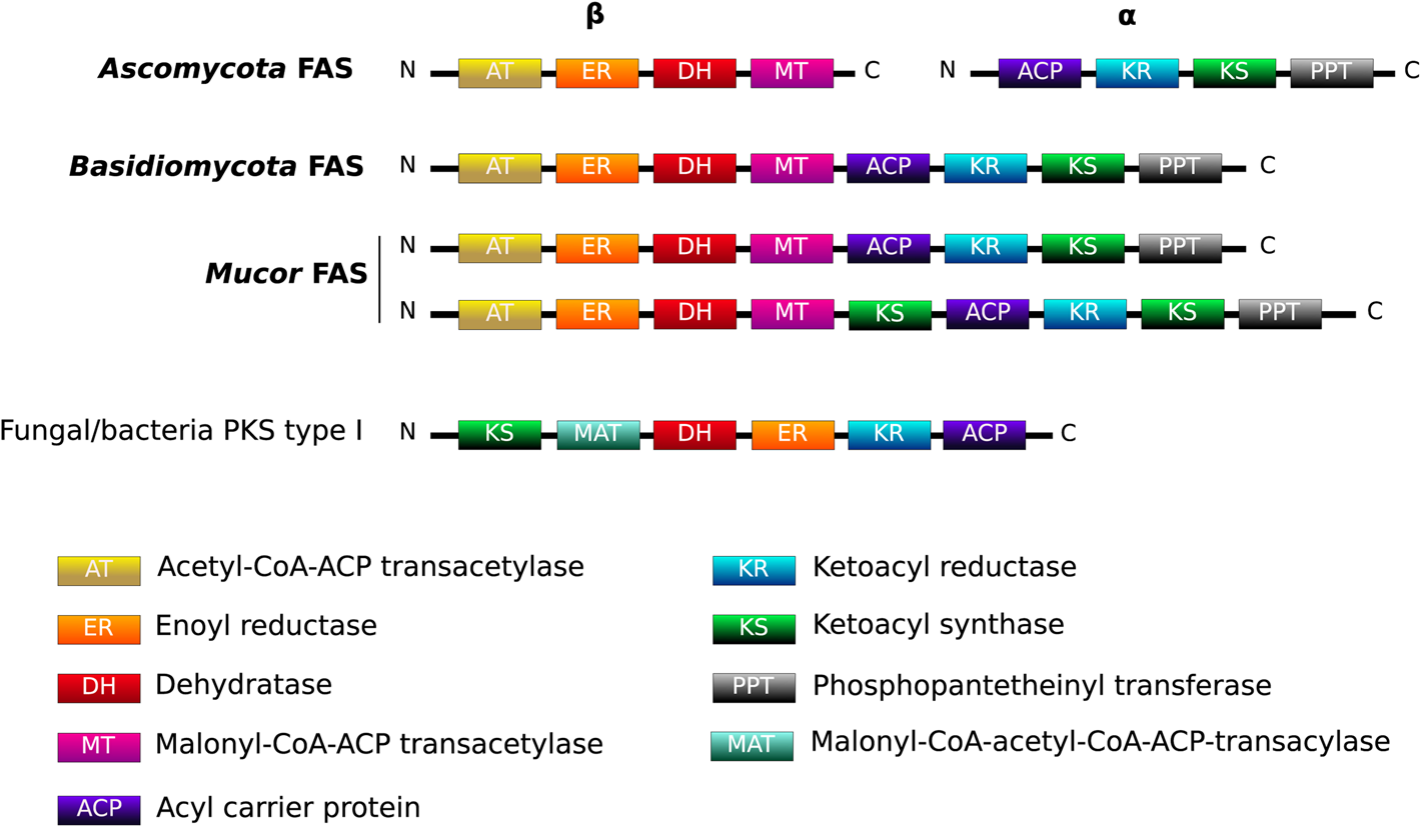
Domain organization of FAS within the studied *Mucor* genomes in comparison to *Ascomycota* and *Basidiomycota* reported FAS organizations

Interestingly, some gene sequences encoding putative PKS can be determined by automated annotation; however, using a manual approach, these genes actually correspond to fatty acid synthases (FAS) type I encoding genes. They were detected in the investigated genomes: three in *M. indicus*, one in *M. fuscus* and *M. lanceolatus* and two in the other genomes. The different domains of these genes were similar in terms of composition and organization to FAS encountered in *Basidiomycota*, while FAS are encoded by two genes in *Ascomycota* (Fig. 5). In *M. indicus*, *M. racemosus* and in *M. velutinosus*, one of the FAS genes held a second KS domain. Recent transcriptomic analyses indicated that these genes were not expressed on PDA medium [23]. Noteworthy, none of the potential secondary metabolism associated genes determined in the different *Mucor* genomes were organized in metabolic clusters.

#### Iron uptake

**Fig. 6:**
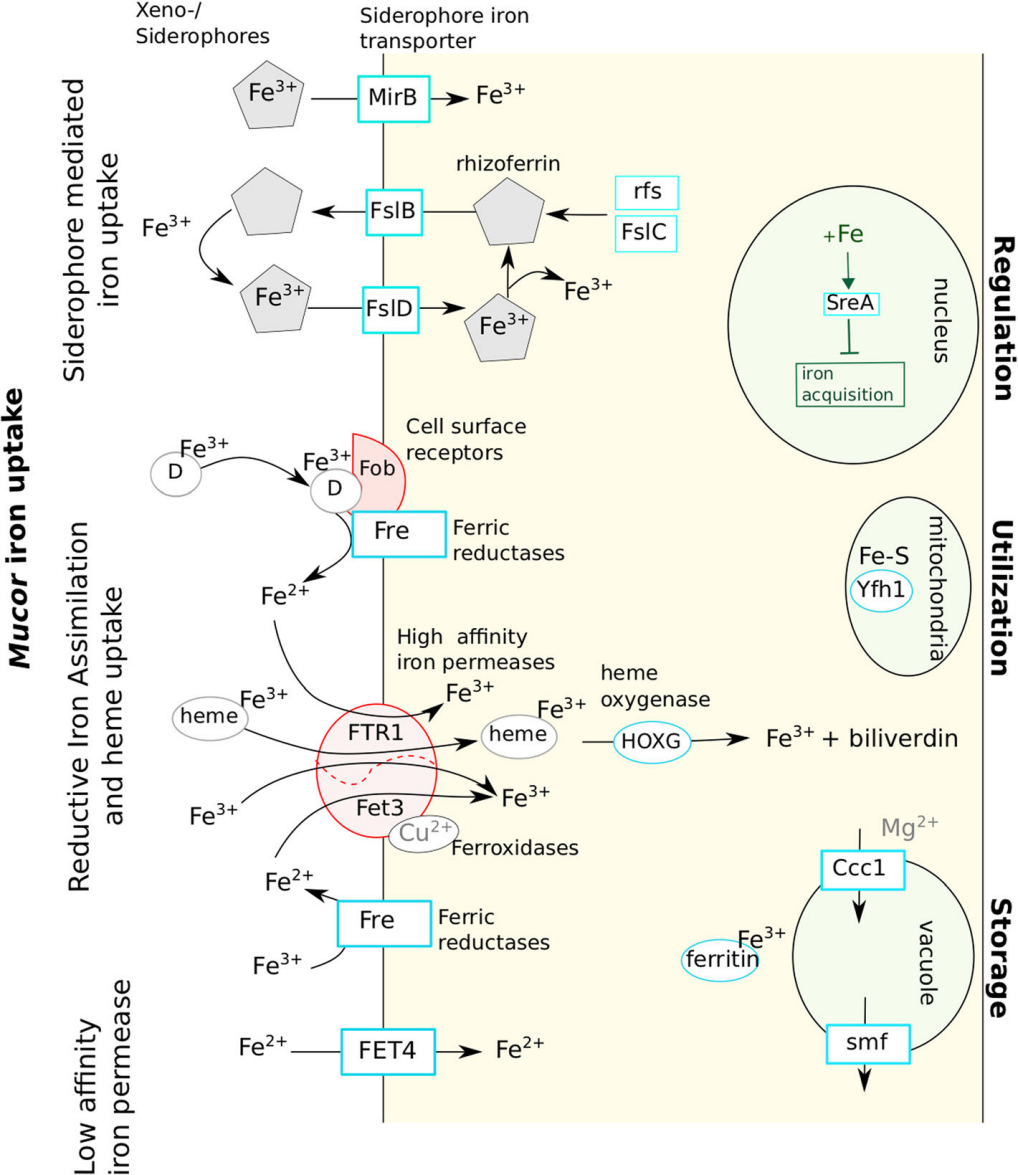
Proposed mechanisms of iron uptake by *Mucor* spp. as well as elements used for iron regulation, utilization and storage. Elements in red were described in literature as important for *Mucor* pathogenicity [13, 16, 32]. D: Deferoxamine

As the involvement of iron uptake in mucormycosis has been reported, the associated genes were studied [30]. Homologs of genes encoding proteins involved in the different iron uptake mechanisms identified so far in fungi [31] were found in the analysed *Mucor* genomes (Table 5; Fig. 6).

**Table 5:**
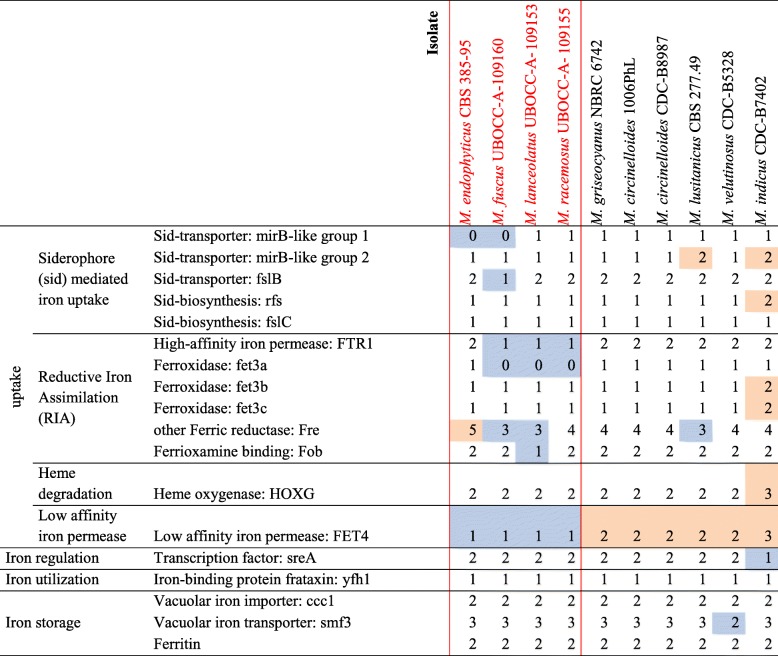
Number of genes involved in iron uptake found in the 10 studied *Mucor*. The four species sequenced in this study are in red. For each gene category, maxima are highlighted in orange and minima in blue. Proteins encoded by the different genes and their role in iron uptake mechanisms are presented in Fig. 6

Regarding the siderophore-mediated iron uptake, genes playing a role in the carboxylate and hydroxamate siderophore synthesis were searched for. At least one ortholog of the *R. delemar rfs* gene, necessary for the carboxylate siderophore rhizoferrin production [33] was found in each *Mucor* species. Other genes that might be involved in this rhizoferrin-mediated iron uptake mechanism were identified based on their homology to the bacterial genes of the *Francisella tularensis* rhizoferrin operon [34]. Homologs of the *FslB* and *FslC F. tularensis* genes were detected in each *Mucor* genome and numerous potential *Mucor* genes belonging the major facilitator family matched to *FslD.* However*, FslA*, *FslE*, *FslF* and the operon regulator *Fur* could not be detected in *Mucor* genomes. Genes involved in hydroxamate siderophore synthesis were not detected but predicted orthologous genes corresponding to the *Aspergillus* MirB siderophore permease (group 1) encoding gene and another gene coding for a MirB-like siderophore permease (group 2) were identified (Table 5).

Regarding the reductive iron assimilation iron uptake, homologous sequences of the gene encoding the *FTR1* high-affinity permease and *fet3* ferroxidase genes were detected (*fet3a* was not detected in *M. fuscus*, *M. lanceolatus* and *M. racemosus* genomes). Except for the FTR1 encoding gene, no gene involved in heme uptake was identified. The FET4 low affinity iron permease encoding gene was identified in the different *Mucor* genomes. When focusing on the iron uptake regulation, homologs to the *SreA* iron uptake repressor gene were detected but no gene involved in activation of iron acquisition pathways such as *HapX* or *Aft* genes were identified. Two ferritin encoding genes of similar lengths were present in each studied species; in each case, the two genes shared 70% similarity and 40% identity. Interestingly, the number of genes involved in iron uptake was always higher in the genome of the opportunistic pathogen *M. indicus* than in the other genomes (Table 5). Indeed, in the *M. indicus* genome*,* (i) for the siderophore pathways, the *rfs* rhizoferrin biosynthesis gene was duplicated as well as a MirB-like siderophore permease encoding gene; (ii) for the reductive iron assimilation (RIA), the *fet3b* and *fet3c* ferroxidase genes were duplicated; (iii) for heme degradation, a supplementary heme oxygenase gene homolog was detected and (iv) for direct Fe^2+^ uptake, three orthologs of the FET4 low affinity permease were identified, whereas two genes were found for the *M. circinelloides* complex and one for the other studied species. Finally, only one copy of the *sreA*-like gene, involved in down regulation of iron acquisition, was found in *M. indicus* whereas at least two copies were found for all other species.

On the contrary, the two cheese-associated species (*M. fuscus* and *M. lanceolatus*) exhibited a reduced number of genes involved in iron uptake within their genomes. *MirB*-like siderophore permease encoding gene was absent from the *M. fuscus* genome whereas one copy of *fslB*-like siderophore permease gene was lost. *M. lanceolatus* lost one copy of the cell surface receptor *fob* gene and of the *FTR1* high affinity permease, both species lacked the *fet3a* ferroxidase genes. The latter genes were also absent from the cheese contaminant *M. racemosus* genome.

#### Antifungal resistance

According to their habitat, fungi can be confronted to chemical biocide. In particular, at the hospital or in agriculture, fungicides are used and fungi can develop resistance. Based on the genome comparison we focused our attention on the antifungal resistance of the studied *Mucor* to triazoles.

**Fig. 7:**
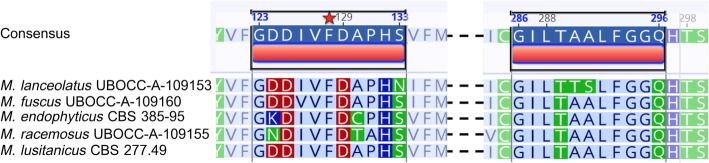
Partial amino acid sequence alignment of lanosterol 14α-demethylase F5 in different *Mucor* isolates. The consensus sequence is represented in blue and polar amino acids appear in green. ML: *M. lanceolatus;* MF*: M. fuscus;* ME: *M. endophyticus;* MR: *M. racemosus;* MLu: *M. lusitanicus*

The deduced aminoacyl sequences of the two lanosterol 14α-demethylase paralogues CYP51 F1 and CYP51 F5 involved in ergosterol biosynthesis were globally well conserved over the *Mucor* genomes with the occurrence of the F128 residue in Helix I of CYP51 F5 which has been reported to be responsible for short-chain azole resistance in *Mucorales* [35]. However, a two-residue substitution (AA to TS at residues 290–291) of Helix I of CYP51 F5 in *M. lanceolatus* was observed (Fig. 7).

According to Caramalho et al., this residue could play a role in the intrinsic resistance against triazoles. These observations raised the question of the impact of these amino acid substitutions on *M. lanceolatus* azole susceptibility. Growth studies on azole supplemented media clearly showed that *M. lanceolatus* was the most susceptible species to both long- (posaconazole) and short-chain (voriconazole) azoles among the tested *Mucor* species (Table 6) [35].

**Table 6:** Growth diameter (cm) of representative *Mucor* isolates on RPMI medium supplemented or not with different concentrations of voriconazole (VCZ) or posaconazole (PCZ)

	VCZ concentration						PCZ concentration					
Isolate	0 mg/L	10 mg/L	50 mg/L	100 mg/L	500 mg/L	1000 mg/L	0 mg/L	10 mg/L	50 mg/L	100 mg/L	500 mg/L	1000 mg/L
*M. endophyticus* CBS 385-95	≥ 5,4	≥ 5,4	≥ 5,4	≥ 5,4	0	0	≥ 5,4	≥ 5,4	≥ 5,4	≥ 5,4	0	0
*M. fuscus* UBOCC-A-109160	≥ 5,4	3,2	0,8	0,2	0,2	0,2	≥ 5,4	3	3	3	1,5	0,2
*M. lanceolatus* UBOCC-A-109153	≥ 5,4	4,5	0	0	0	0	≥ 5,4	0	0	0	0	0
*M. racemosus* UBOCC-A-109155	≥ 5,4	≥ 5,4	≥ 5,4	≥ 5,4	3,2	0,5	≥ 5,4	≥ 5,4	≥ 5,4	≥ 5,4	≥ 5,4	≥ 5,4
*M. lusitanicus* CBS 277.49	≥ 5,4	≥ 5,4	≥ 5,4	≥ 5,4	0,3	0,3	≥ 5,4	≥ 5,4	≥ 5,4	≥ 5,4	≥ 5,4	≥ 5,4

## Discussion

Although benefiting from a growing interest due to their involvement in mucormycosis but also their biotechnological potential, a restricted number of *Mucor* genomes is available to this date, leading to scarce whole genome comparative studies [13, 15, 36]. These comparative studies mainly focused on human/clinical environments species. Yet, only a handful of *Mucor* species are known to cause human infections [2]. In this context, this study expanded the range of *Mucor* genomes available by including genomes from species collected from non-clinical environments. In particular, *M. endophyticus* was only reported as a wheat endophyte [27] and *M. lanceolatus* was, so far, only collected from cheeses [26].

The determined phylogeny, based on a large set of orthologous genes (51), that integrated the four genome sequences obtained in this study is partially concordant with published phylogenies. In particular it confirmed the phylogenetic relationships amongst different members of the *M. circinelloides* complex [4] but was non-concordant with the latest published phylogeny concerning the phylogenetic position of *R. microsporus* CDC-B9738 and CDC-B9645 isolates, as they appeared in a sister group of the *M. circinelloides* complex and closely related to *M. racemosus*. This was observed both in the present study and that of Chibucos et al. (76 analysed orthologs) while they clustered into the *R. microsporus* clade with *M. circinelloides* species as an outgroup in Gryganskyi et al. study (192 orthologues analysed) [13, 25]. Topologies may vary depending on the selected genes and on the reconstruction pipeline. The contrasted placements of the 2 *R. microsporus* isolates, which harbour larger-sized genomes (Fig. 2c), among the different studies may arise from the whole/partial-genome duplication events and/or hybridization observed in their genomes [25]. As a confirmation, when considering gene families involved in secondary metabolism and iron acquisition (which were investigated in this study by comparative genomics approaches), *R. microsporus* CDC-B9738 and CDC-B9645 genes were often found duplicated. Phylogenetic reconstructions performed using the duplicates yielded incongruent topologies with either CDC-B9738 or CDC-B9645 isolates clustering with *R. delemar* clade or alternatively with *M. racemosus* clade suggesting a possible hybrid genomic content for these two isolates.

Whatever the phylogenetic placement of the studied species or their proposed habitat/lifestyle, the current study revealed that the gene features among *Mucor* species (gene number, size, exon length etc.) were globally conserved. However, as indicated by the lack of macro- and micro-synteny (data not shown), species within this genus experienced extensive genomic rearrangements. These rearrangements can be partially explained by transposable elements (TE), which displayed high degree of diversity within the available genomes and have already been reported to have a major role in fungal genomic diversity and genome evolution [37].

The average genome size of the *Mucor* species analysed in this study (39 Mb) is congruent with what is observed at the scale of the *Mucoromycotina* subphylum (38 Mb) and also in *Ascomycota* (37 Mb). The gene number (9997 to 12,571) is also in concordance with the gene numbers observed in *Mucoromycotina* and *Ascomycota* [38]. The core genome detected in this study includes approximately 6000 genes. The species-specific genes and the genes shared by species with similar lifestyles (data not shown) mostly encode proteins with unknown functions. They are thought to determine specific traits, such as adaptation to different environmental niches or preferential colonization of certain habitats, and determining their function would be of special interest.

As osmotrophic organisms, fungi depend, for gaining nutrients, on lytic enzyme secretion to externally break down surrounding resources prior to absorption of the catabolized products [39]. Depending on the lifestyle (saprotrophic, pathogenic or mutualistic) and the habitat, the way of gaining nutrients and energy may vary. In particular qualitative and quantitative differences in terms of CAZymes can be potent reporters of fungal lifestyle [38, 40–42]. In the present study, diverging CAZyme repertoires among species or species groups sharing the same ecology were identified. The genome of *M. endophyticus* CBS385–95, isolated from wheat leaves and described as an endophyte, shares similar traits with genomes of other fungal endophytes. It harbours a low number of catabolic CAZymes (CBM, GH and PL) and esterase (CE) genes [43] including genes coding for plant cell wall degrading enzymes, such as the GH5, GH3 and GH9 fungal cellulases encoding genes. This observation suggests that *M. endophyticus*, like other fungal endophytes and ectomycorrhizal fungi, is indeed adapted to intercellular growth and to the avoidance of the plant immune system [43, 44]. The two species almost only isolated from cheeses and involved in cheese ripening (*M. fuscus* and *M. lanceolatus*) also displayed, to a lesser extent than *M. endophyticus*, a reduced repertoire of carbohydrate active enzyme encoding genes. The reduction was less significant for the GT encoding genes whose roles are not directly connected to the external environment. It could be hypothesized that this reduced set of catabolic CAZymes is an imprint of specialization to a less ubiquitous lifestyle and an adaptation to the cheese environment. In contrast, the thermophilic opportunistic pathogen *M. indicus* harbours the largest set of CAZyme genes. This trait is not shared by *M. circinelloides*, a species that may cause mucormycosis. This could be related rather to the high capacity of *M. indicus* to degrade plant materials [45] than to an opportunistic pathogenic lifestyle. However, it is worth noting that *M. indicus* possesses a much larger protease repertoire compared to the other *Mucor* species including the *M. circinelloides*. Given that proteases [46, 47] were found clearly correlated to pathogenicity in *Ascomycota* opportunistic pathogens one might expect a higher pathogenicity potential of *M. indicus* over *M. circinelloides*.

Examination of secondary metabolic pathways can also shed light on fungal ecology and adaptation to a particular lifestyle [48]. In this study, we investigated more specifically genes that are essential to most of the secondary metabolite biosynthesis (PKS, NRPS, DMATS …) but also genes involved in iron uptake which have been shown to be fundamental for fungal virulence in opportunistic pathogens [49, 50] and for cheese colonizing microorganisms given that cheese is a strongly iron-restricted medium [51]. The search for genes involved in secondary metabolite biosynthesis are often initiated by the search for PKS, NRPS or DMATS genes clustered with additional genes involved in the considered biosynthesis pathway and known as BGC (biosynthetic gene cluster). In the framework of this study, no typical BGCs involved in secondary metabolism, including a PKS, FAS, NRPS, NRPS-like gene or terpene synthesis related genes, were detected within the studied *Mucor* genomes. Still, secondary metabolites pathways have been characterized in *Mucorales* [3] and different genes involved in secondary metabolism were identified in the present and previous studies. The apparent absence or at least scarcity of BGCs within the genus *Mucor* (and possibly at a broader scale within the *Mucoromycotina*) might appear enigmatic since the genus encompasses species with contrasting lifestyles similar to what exists in *Ascomycota* in which BGCs are abundant. This raises the question why the apparent selective advantage conferred by BGCs to higher fungi would not apply to *Mucor* species. The answer to this question might lie in some ecological specificities of the *Mucorales* which are considered as ruderal species avoiding stress and competition [52, 53] or to structural specificities such as their coenocytic structure [54], and may shed new light regarding the BGC evolution in eukaryotes. All *Mucor* genomes investigated in this study included a NRPS gene which role has still to be determined as well as different genes encoding for enzymes associated with terpene biosynthesis. As noticed by Voigt et al. who analysed different *Mucor* genomes, no PKS genes but genes with a typical structure and domain order of Type I fatty acid synthases (FAS) were detected in the different *Mucor* genomes investigated in the present study [3]. These *Mucor* FAS have their domains allocated within a single gene as in *Basidiomycota* and not over two genes as in *Ascomycota* (Fig. 5) [55, 56]. This result supports the hypothesis of a primordial contiguous FAS gene encoding the entire FAS [55]. Most of the *Mucor* genomes analysed here include two putative FAS genes, but a single FAS gene was found in the cheese-related species *M. lanceolatus* and *M. fuscus*, and up to three genes were found in the opportunistic pathogen *M. indicus*. Whatever the number of FAS genes included in the genome, only one FAS was found expressed when the isolate was cultivated on PDA medium [23]. It could be hypothesized that the additional FAS genes have lost their functionality or display a different role since FAS gene are expected to be expressed in such conditions to participate to the fatty acid synthesis for membrane synthesis and energy storage [56]. Whether or not this additional FAS might be involved in secondary metabolisms like specialized secondary metabolism FAS [57] has to be determined.

Among the secondary metabolites, terpenes play a role in natural pigment synthesis such as the carotenoid biosynthesis [3]. Carotenoid synthesis has been described in *M. circinelloides* and in particular in overproducing isolates [3, 58, 59]. Although the different species within the *Mucoromycotina* subphylum are expected to produce and accumulate large amount of carotenoids, differences might exist among species [60]. Terpenes play also an important role in flavour production which is an important trait for cheese-ripening fungi that could have been under human-directed selection in species used for cheese making [61]*.* This study indicated that the main genes involved in terpene biosynthesis were conserved among the analysed *Mucor* species without any important differences in terms of gene number with the exception of GGPP genes for which the number is lower in the endophytic species as well as in *M. griseocyanus* and in the *M. indicus* opportunistic pathogen.

Iron is an essential nutrient involved in variety of cellular processes such as respiration, oxidative stress and amino acid synthesis [62]. Iron uptake has been described as a virulence factor for pathogenic fungal isolates [49, 50] and of primary importance in cheese microorganisms since cheese is a highly iron-depleted medium [51]. In fungi, four different iron uptake mechanisms have been described so far: (i) siderophore mediated Fe^3+^ uptake, (ii) reductive iron assimilation (RIA), (iii) heme uptake and (iv) direct Fe^2+^ uptake [49] (Fig. 6). Homologs of genes coding for proteins participating to the four mechanisms were found in the analysed *Mucor* genomes. These results suggest that the different *Mucor* species investigated here rely on carboxylate siderophore rhizoferrin as it is the case for *M. circinelloides* CBS 277.49 and *R. delemar* [33]. This type of siderophore is not used by *Ascomycota* and *Basidiomycota*, and would thus be a specificity of early diverging fungi in the fungal kingdom. Noteworthy, rhizoferrin encoding gene sequences have only been described in bacterial genomes so far [32, 63] and coding sequences pertaining to the rhizoferrin operon in *F. tularensis* were used here to search for homologs in *Mucor* spp. In *F*. *tularensis*, genes involved in rhizoferrin synthesis and transport are located in an operon regulated by the *Fur* gene [34]. Based on these results, new candidate genes involved in rhizoferrin synthesis, import and export are proposed in the present study (Fig. 6). Although no actual evidence of *Mucor* ability to produce hydroxamate siderophores was detected, some of these siderophores could be used by *Mucor* species as xeno-siderophores as suggested by the presence of *mirB*-like siderophore transporter genes in some of the *Mucor* genomes. It is worth noting that the bacterial siderophore deferoxamine is also used by *Mucor* spp. as xeno-siderophores [64].

Interestingly, the three isolates sampled from cheese presented a reduced number of genes related to iron acquisition compared to the other isolates. Indeed, the genomes of these isolates lack a FTR1 gene copy as well as the *fet3a* gene. It could be hypothesized that the latter genes would have a specific role in *Mucor* pathogenicity since *R. delemar* mutants with FTR1 reduced gene copies or with decreased FTR1 expression had reduced virulence in a deferoxamine-treated mouse model of mucormycosis [30]. The *fet3a* gene appeared as the less important among the *fet3* genes regarding *M. circinelloides* pathogenicity, but inactivation of two *fet3* genes led to a reduced virulence [17] and the loss of *fet3a* led to an increased sensibility to the mutations on *fet3b/fet3c* in terms of fungal pathogenicity. Furthermore, one copy of the ferroxamine binding (Fob) cell surface protein gene is absent in the *M. lanceolatus* genome, the production of this protein being required for full virulence of *R. delemar* in a deferoxamine-treated mouse model of mucormycosis [14]. *M. fuscus* also lacks *MirB*-like and one copy of *fslB*-like siderophore permease genes, which might reduce its potential to acquire iron. These results provide possible evidence of safety of these food-related species although the number of studied isolates is too low to definitely assert it. On the contrary, *M. indicus*, a species that is considered as the most threatening opportunistic human and animal pathogen amongst the *Mucor* species [7] harbours an increased set of genes involved in iron uptake which might be an asset to its opportunistic pathogenic lifestyle.

Susceptibility to antifungal drugs, and in particular to azole antifungal agents which are widely used for mucormycosis treatments as well in agriculture [35, 65], is of interest as it may vary in *Mucor* species according to their specific habitats, e. g. cheese isolates are probably less exposed to antifungals and antifungal resistance is unlikely to offer a selective advantage to non-pathogens. Azole drugs inhibit lanosterol 14α-demethylase (LDM) thus blocking ergosterol biosynthesis and resulting in a build-up of toxic sterols [66]. In *Mucorales*, two lanosterol 14α-demethylase paralogues CYP51 F1 and F5 coexist with a substitution in CYP51 F5 at residue 128 responsible for innate resistance against short-tailed triazoles, and a V to A substitution at residue 291 of CYP51 F5 also potentially playing a role [35]. Interestingly, we found that *M. lanceolatus*, only isolated so far from cheese, did not harbour the V to A substitution at residue 291 of the CYP51 F5 predicted protein but bears instead a unique two aminoacyl substitution (AA to TS) at residues 290 and 291 (in consensus sequence). Azole tests performed in this study demonstrated that these substitutions were associated with a notable higher susceptibility to both short- and long-tailed azoles.

## Conclusions

In conclusion, this study expanded the range of *Mucor* genomes available by including genomes from species with contrasted lifestyles represented by isolates collected from non-clinical environments (more specifically, cheese and plant). In addition to yield a better overview of the *Mucor* pan-genome, the expanded range of genomes allowed identifying contrasting features that could contribute to habitat and niche adaptation although distinguishing divergences due respectively to evolutive adaptation or to non-ecologically based evolutionary forces may appear difficult given that the different taxa did not diverged recently. The obtained data will allow further investigating the link between genetic and biological data, especially in terms of niche or habitat adaptation.

## Materials and methods

### Biological material

The genomes of 12 representative isolates were investigated in the present study (Table 7). Four of them were sequenced in the framework of this study while the eight others were publicly available [12, 13, 28]. *M. fuscus* UBOCC-A-109160, *M. lanceolatus* UBOCC-A-109153, *M. endophyticus* CBS 385–95 (UBOCC-A-113049) and *M. racemosus* UBOCC-A-109155 used for genome sequencing were cultivated in the dark at 25 °C on PDA solid medium (Difco Laboratories, Detroit, Michigan). Spore suspensions of each isolate were produced as previously described by Morin-Sardin et al. [7]. Concentrations were adjusted to 10^7^ to 10^8^ spores·mL^− 1^ prior to storage at − 80 °C until use. For genomic DNA extraction, each of the studied monospore isolates was grown on PDA solid medium at 25 °C for 7 days.

**Table 7:** List of isolates used in this study and their reported habitat. The four isolates sequenced in the context of this study are in red

Species	Isolate	Isolation source	Reported habitat of the species	Reported habitat references	Genome reference or accession
	CBS 385–95 (UBOCC-A-113049)	*Triticum aestivum*, leaves	Plant endophyte	[27]	This study
	UBOCC-A-109160	Cheese	Cheese	[26]	This study
	UBOCC-A-109153	Cheese	Cheese	[26]	This study
	UBOCC-A-109155	Cheese	Cheese, yogurt, walnuts, sausages, grassland soil, decaying vegetables, human	[24, 26, 67]	This study
*M. griseocyanus*^a^	NBRC 6742	Unknown	Vegetable, tanned sole leather	[68]	BBKB00000000
*M. circinelloides* f. *circinelloides*	1006PhL	Skin of a healthy human	Cheese, sufu starter, decaying vegetables, human, soda, air, soil, dung, sediment	[22, 24, 26, 69]	[29]
*M. circinelloides*	*CDC-B8987*	Human BL line			[13]
*M. lusitanicus*^b^	CBS 277.49 (UBOCC-A-108085)	Unknown			[12]
*M. velutinosus*^c^	*CDC-B5328*	Human: nasal			[13]
*M. indicus*	*CDC-B7402*	Human: unknown	Human, dung, *Dioscorea tuber*, sorghum malt	[24, 70, 71]	[13]
*R. microsporus (formerly M. racemosus*^c^*)*	*CDC-B9645*	Clean room floor	Human, dust, sorghum malt, stored cereals	[13, 24]	[13]
*R. microsporus (formerly M. racemosus*^c^*)*	*CDC-B9738*	Human abdomen			GCA_000697275.1

*CBS (Centraalbureau voor Schimmelcultures, Netherlands), NBRC (Biological Ressource Center, NITE), NRRL (Agricultural Research Service Culture Collection, USA), CDC (Centers for disease Control and Prevention) and UBOCC (University of Brest Culture Collection)*

^a^Originally *M. ambiguus* NBRC 6742. ^b^Originally *M. circinelloides* CBS 277.49. These isolates were reassigned according Wagner et al. [4]. ^c^Initially referenced as *Mucor racemosus* [13] but later assigned to *Rhizopus microsporus* [25]

### Species assignment within *M. circinelloides* complex

Species assignment of isolates pertaining to the *M. circinelloides* complex was performed using a Maximum Likelihood phylogenetic reconstruction based on a *cyclopropane-fatty-acylphospholipidsynthase* gene (*cfs*) sequence alignment including *cfs* sequences available in Wagner et al. [4].

### Genome sequencing and assembly

Genomic DNA from *M. fuscus, M. racemosus, M. lanceolatus* and *M. endophyticus* was extracted from fresh mycelium, following the genomic DNA extraction method as outlined by the 1000 Fungal Genomes Project (http://1000.fungalgenomes.org/home/protocols/high-quality-genomic-dna-extraction/#Fulton1995) which is based on a protocol by Spanu et al. [72]. with an optional step using Qiagen genome-tips (Qiagen). Due to the low efficiency of the CTAB method for *M. lanceolatus*, genomic DNA of this isolate was also extracted following the protocol developed by Cheeseman et al. with a purification by a cesium chloride gradient with DAPI [73].

Genomes were sequenced with Illumina technology (San Diego, CA) at different sequencing facilities (Additional file 5: Table S2). For each of the four species, DNA were paired end sequenced (read length 2 × 100 bp, insert size 500 bp). An additional mate pair sequencing was performed for *M. lanceolatus* and *M. endophyticus* (read length 2 × 100 bp, insert size 9–12 kb). Sequences were quality checked with FastQC [74]. Adaptors were removed, reads were quality trimmed (bases kept had a phred score above 25) and reads shorter than 20 bp were dropped with Cutadapt [75]. Mate pair reads of *M. lanceolatus* were mapped with STAR [76] on a preliminary version of the assembly (by providing only *M. lanceolatus* paired end data to CLC Genomics Workbench -CLCbio, Seoul, Korea-). Mate pair reads separated by less than 500 bp and oriented in forward-reverse were dropped. This new set of reads was used in further *M. lanceolatus* assemblies. *M. lanceolatus* and *M. endophyticus* genomes were assembled using Velvet [77] (option “shortMatePaired”, k-mer of 67 for *M. lanceolatus* and k-mer of 85 for *M. endophyticus*), while *M. racemosus* and *M. fuscus* genomes were assembled with SOAPdenovo [78]. Genome assembly quality was checked with BUSCO v3 [79] using the fungal dataset and *Rhizopus* Augustus training.

### Genome annotation of the four newly sequenced genomes

Genome assembly scaffolds were annotated using combinations of ab initio predictors, RNAseq data support and homology search. As for ab initio predictors, Genemark-ES [80], with self-training, and Augustus [81], with *Rhizopus* training available within the Augustus tool, were used. RNAseq transcripts were extracted and sequenced as previously described in Lebreton et al. and reconstructed using two methods [23]: (i) by mapping RNAseq reads on genome with STAR [82] and reconstructing transcripts with Cufflinks [83], and (ii) by de novo transcript reconstruction with Trinity [84] and mapping the obtained transcripts on the genomes with GMAP [85]. Predicted proteins of *M. lusitanicus* [12], *R. delemar* [9] and *P. blakesleeanus* [12] were searched on genomes with Exonerate [86]. Consensus gene models were generated from all predictions by EVidenceModeler [87].

The obtained gene predictions were functionally annotated as follows: transmembrane domains were predicted with TMHMM [88], peptide signal with SignalP v4 [89] and Pfam domains with HMMER [90] using the PFAM-A database [91]. Sequence homologies were searched using tBLASTx and BLASTp [92] (with an e-value threshold inferior to 10^− 5^, against Swissprot-Uniprot and Uniref90 databases as well as *M. lusitanicus* CBS 277–49, *R. delemar* RA-99880 and *P. blakesleeanus* NRRL1555 filtered proteins obtained from the JGI platform [93]. EC numbers were predicted using PRIAM [94] and were transferred from homology researches. GO terms were transferred from homology search. Gene names were assigned with AHRD (Automated Assignment of Human Readable Descriptions) available on Github (https://github.com/groupschoof/AHRD).

Non-coding RNA were predicted with tRNAscan-SE [95], RNAmmer [96] and Infernal [97] using the Rfam database [98]. The obtained data were integrated in an instance of the genome viewer Apollo [99] allowing experts to validate gene prediction quality and perform manual curation.

### Complementary annotation of the full set of genomes

Transposable elements (TE) were annotated using the REPET pipeline [100] that includes a de novo prediction and TE classification [101]. Carbohydrate-active enzymes were searched using dbCAN2 [102] based on sequences available in the CAZy database [103] with HMMER (E-Value <1e^− 15^, coverage > 0.35), DIAMOND [104] (E-Value <1e^− 102^) and Hotpep [105] (Frequency > 6.0, Hits > 2.6). Only annotations predicted by at least two different tools were subsequently considered. Peptidases and their inhibitors available in the MEROPS database [106] were searched using BLASTp (E-Value 10^− 5^).

### Phylogenetic reconstruction

Predicted proteomes of the 12 studied isolates, as well as those of *R. delemar* and *P. blakesleeanus* (both latter species being considered as outgroups), were compared based on sequence similarity to identify orthologous proteins using the Orthofinder v.2.2.0 software [107] (E-value 10^− 5^, inflation 1.5). The 64 obtained single copy orthologs were selected to reconstruct the phylogeny of the studied species. Multiple alignment was inferred using PRANK v.170427 [108], run with default settings. Spuriously aligned regions were excluded with TrimAl v1.4.r15 [109] with a 0.2 gap threshold. Based on the alignments, 13 orthogroups were manually discarded due to low percentage of identical sites or high number of gaps among orthologs. Subsequent alignments were concatenated in a supermatrix of 23,398 sites. This matrix was used to reconstruct the species tree in one hand using Bayesian Monte Carlo Markov Chain (MCMC) samples with PhyloBayes v3.3 MCMC samplers [110] with a CAT+GTR model and 3 chains and in the other hand by Maximum Likelihood inference with RAxML PTHREADS v. 8.2.9 [111], with a partitioned LG + G model, in which each data partition represented a single input gene family. A bootstrap analysis with 100 replicates under the same model was performed in RAxML in order to assess tree branch support. To confirm the obtained phylogenetic position of the 2 *R. microsporus* isolates, an expanded species tree including 11 other sequenced *Mucoromycotina* (25 isolates overall) was reconstructed as follows. The 29 single copy genes families containing at least 20 isolates were independently aligned with PRANK v.170427 and trimmed with TrimAl v1.4.r15. For each gene family a gene tree was reconstructed using RAxML with the auto-detection of the best amino-acid model and a bootstrap analysis of 100 samples. The species tree was then reconstructed using the 29 gene trees with Clann v4.2.2 using a bootstrap analysis of 100 samples [112]. The obtained RAxML tree of the main species was used to estimate the divergence time between species with the Langley-Fitch method with r8s v1.8 [113] by calibrating against the assessed origins of *P. blakesleeanus* and *R. delemar* at 468 MY [114].

### Evolution of genes families

Based on OrthoFinder results and the obtained ultrametric tree, expansion and contraction of gene families were reconstructed with CAFE v4 [115]. Birth and death parameters were estimated independently using orthologous groups containing less than 75 genes per isolate. The analysis was done on all isolates except *R. microsporus* CDC-B9645 and *R. microsporus* CDC-B9738. Rapidly evolving families were predicted by CAFE using the Viterbi algorithm.

### Secondary metabolism related genes

Secondary metabolism associated genes (polyketide synthase -PKS-, non-ribosomal peptide synthetase -NRPS-, terpene synthase -TPS-, dimethylallyl tryptophan synthase -DMATS-) and other genes potentially involved in adaptation to the environment (e.g. genes related to iron acquisition and to antifungal resistance) were searched in each species. Gene cluster associated with secondary metabolites were searched with antiSMASH v.4 fungal version (FungiSMASH) [116] and SMURF [117].

### Antifungal susceptibility

*M. lusitanicus*, *M. endophyticus*, *M. fuscus*, *M. lanceolatus* and *M. racemosus*, as well as *Byssochlamys fulva* UBOCC-A-101005 (as a positive control), were grown in 5.4 cm Petri dishes on solid RPMI medium (Gibco BRL, Gaithersburg, MD) with 2% glucose and different concentrations of azole antifungal compounds: 0 mg.l^− 1^, 10 mg.l^− 1^, 50 mg.l^− 1^, 100 mg.l^−^ 1, 500 mg.l^− 1^ and 1000 mg.l^− 1^ of voriconazole (VCZ) (OHRE Pharam, Tours, France) or posaconazole (PCZ) (MSD France, Courbevoie, France), respectively. Each antifungal concentration was assayed in triplicates. Growth diameters were measured after 7 days.

### Availability of supporting data

Raw sequence data were deposited at the European Nucleotide Archive (ENA) (http://www.ebi.ac.uk/ena/data/view/PRJEB30975) and genome assemblies, annotations and genome browser are available on the ABiMS platform (http://application.sb-roscoff.fr/project/mucor/) as well as on the JGI Mycocosm platform (https://genome.jgi.doe.gov/programs/fungi/index.jsf). Noteworthy, functional annotations found on the Mycocosm platform differ from the ones exploited here since the structural annotation was functionally re-annotated by the pipelines associated with the Mycocosm platform (https://genome.jgi.doe.gov/Mucrac1, https://genome.jgi.doe.gov/Mucend1, https://genome.jgi.doe.gov/Mucfus1, https://genome.jgi.doe.gov/Muclan1). All the genome sequences compared to the genomes sequenced in the present study were found in NCBI Genome except for *M. lusitanicus* genome available at the Mycocosm platform.

## Supplementary information

**Additional file 1: Figure S1.** Phylogenomic tree for the genome of 25 *Mucoromycotina*. The tree was reconstructed using Clann based on 29 genes trees. Each tree corresponded to one of the single copy gene families included at least 20 of the 25 isolates investigated. Bootstrap supports are indicated under the branch.

**Additional file 2: Figure S2.** Representation of the TE coverage in each genome depending on the TE category (classification of Wicker et al.) [118]. The four species sequenced in this study are in red. DHX: Helitron transposon. DTX: TIR transposon. DXX-MITE: unknown non-autonomous transposon, MITE-like. noCat: potential transposable element that could not be identified. RIX: LINE retrotransposons. RLX: LTR retrotransposon. RXX unknown retrotransposon. RXX-LARD: unknown non-autonomous retrotransposon, LARD-like. RXX-TRIM: unknown non-autonomous retrotransposon, TRIM-like.

**Additional file 3: Figure S3.** Number of CAZymes in the different fungal phyla. Major CAZymes classes are shown separately, Auxiliary redox enzymes (AA), Carbohydrate-Binding Modules (CBM), Carbohydrate Esterases (CE), Glycoside Hydrolases (GH), Glycoside Transferases (GT) and Polysaccharide Lyases (PL).

**Additional file 4: Table S1.** Number of identified CAZyme encoding genes involved in the degradation of plant cell wall components (Cellulose active, Hemicellulose active and Pectin active). *The presence of genes has been confirmed with manual annotation*.

**Additional file 5: Table S2.** Extraction and sequencing information corresponding to the four newly sequenced *Mucor* isolates

## Data Availability

Raw sequence data were deposited at the European Nucleotide Archive (ENA) (http://www.ebi.ac.uk/ena/data/view/PRJEB30975), genome assemblies and annotations are available on the ABiMS platform (http://application.sb-roscoff.fr/project/mucor/).

## Abbreviations

CAZymes: Carbohydrates Active enZymes
CYP51: 14α-demethylase paralogues
DMATS: DiMethylAllyl Tryptophan Synthase
FAS: Fatty Acid Synthase
NRPS: NonRibosomal Peptide Synthetase
PCZ: Posaconazole
PKS: Polyketide Synthase
RIA: Reductive Iron Assimilation
SSP: Small secreted protein
TPS: Terpene Synthase
VCZ: Voriconazole
