# Supplementary figures and images for: Comparative genomics applied to Mucor species with different lifestyles
Source: BMC Genomics. 2020 Feb 10;21:135. doi: 10.1186/s12864-019-6256-2 (PMC7011435; doi:10.1186/s12864-019-6256-2)

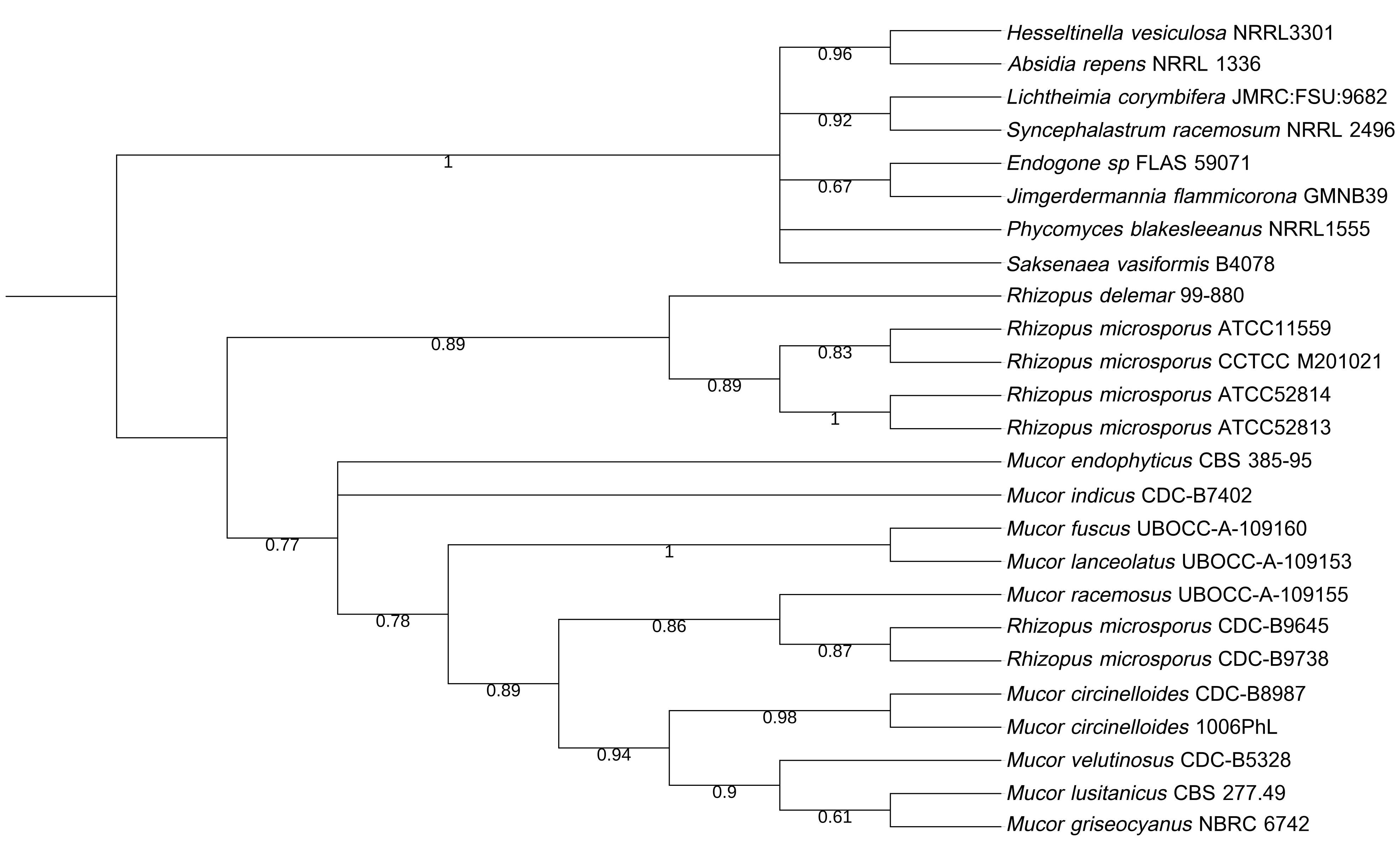

Supplement: Supplementary file 1 — Additional file 1: Figure S1. Phylogenomic tree for the genome of 25 Mucoromycotina. The tree was reconstructed using Clann based on 29 genes trees. Each tree corresponded to one of the single copy gene families included at least 20 of the 25 isolates investigated. Bootstrap supports are indicated under the branch. [file 12864_2019_6256_MOESM1_ESM.tiff]

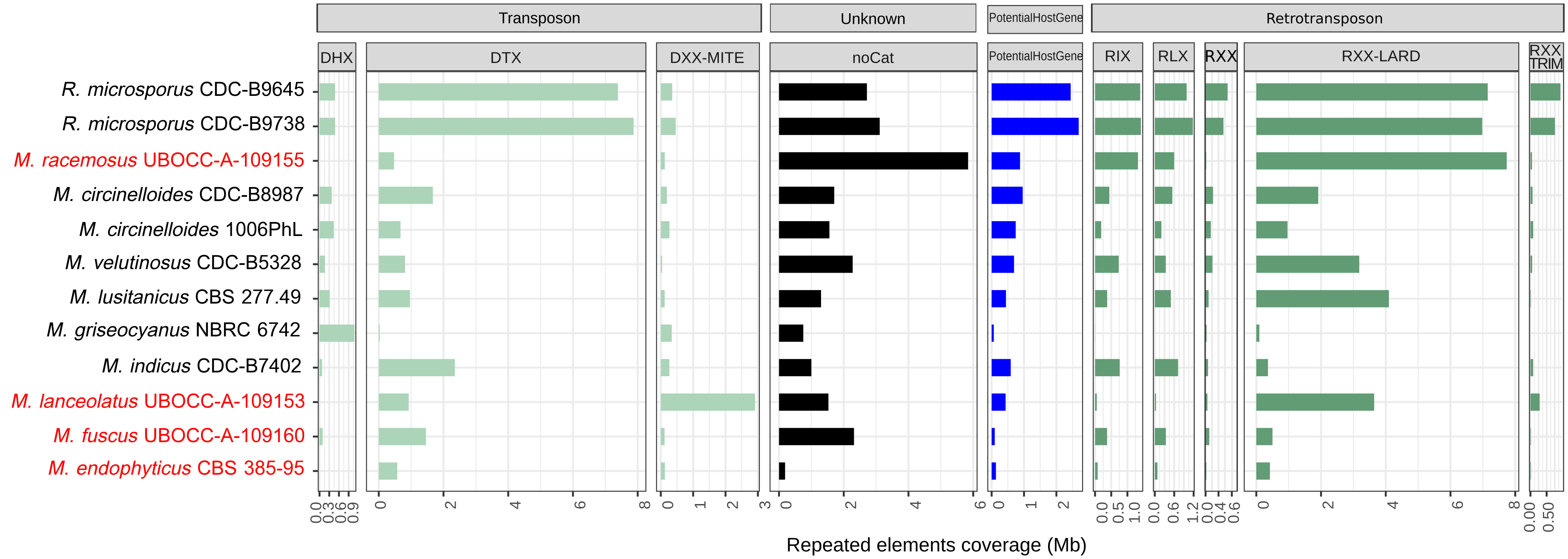

Supplement: Supplementary file 2 — Additional file 2: Figure S2. Representation of the TE coverage in each genome depending on the TE category (classification of Wicker et al.) [118]. The four species sequenced in this study are in red. DHX: Helitron transposon. DTX: TIR transposon. DXX-MITE: unknown non-autonomous transposon, MITE-like. noCat: potential transposable element that could not be identified. RIX: LINE retrotransposons. RLX: LTR retrotransposon. RXX unknown retrotransposon. RXX-LARD: unknown non-autonomous retrotransposon, LARD-like. RXX-TRIM: unknown non-autonomous retrotransposon, TRIM-like. [file 12864_2019_6256_MOESM2_ESM.tiff]
