# Supplementary material for: Comparative genomics applied to Mucor species with different lifestyles
Source: BMC Genomics. 2020 Feb 10;21:135. doi: 10.1186/s12864-019-6256-2 (PMC7011435; doi:10.1186/s12864-019-6256-2)
Supplement: Supplementary file 4 — Additional file 4: Table S1. Number of identified CAZyme encoding genes involved in the degradation of plant cell wall components (Cellulose active, Hemicellulose active and Pectin active). The presence of genes has been confirmed with manual annotation. [file 12864_2019_6256_MOESM4_ESM.docx]

**Table S1 :** **Number of identified CAZyme encoding genes involved in the degradation of plant cell wall components** (Cellulose active, Hemicellulose active and Pectin active). *The presence of genes has been confirmed with manual annotation*.

| Isolate | Cellulose | | | | Hemicellulose | | | | | Pectin | |
| --- | --- | --- | --- | --- | --- | --- | --- | --- | --- | --- | --- |
|  | GH3 | GH5 | GH9 | GH45 | CE16 | GH5 | GH29 | GH31 | GH35 | GH28 | PL14 |
| *M. endophyticus* CBS 385-95 | 3 | 4 | 3 | 1 | 0 | 4 | 2 | 3 | 0 | 0 | 2 |
| *M. fuscus* UBOCC-A-109160 | 4 | 4 | 2 | 2 | 1 | 4 | 2 | 3 | 1 | 1 | 1 |
| *M. lanceolatus* UBOCC-A-109153 | 3 | 4 | 2 | 2 | 0 | 4 | 2 | 3 | 1 | 1 | 2 |
| *M. racemosus* UBOCC-A-109155 | 7 | 5 | 4 | 1 | 1 | 5 | 2 | 7 | 1 | 1 | 2 |
| *M. griseocyanus* NBRC_6742 | 6 | 6 | 4 | 1 | 1 | 6 | 2 | 4 | 1 | 2 | 2 |
| *M. circinelloides* 1006PhL | 6 | 6 | 4 | 1 | 0 | 6 | 2 | 6 | 1 | 1 | 2 |
| *M. circinelloides* CDC-B8987 | 6 | 5 | 4 | 1 | 0 | 5 | 2 | 6 | 1 | 1 | 2 |
| *M. lusitanicus* CBS 277.49 | 5 | 6 | 4 | 0 | 0 | 6 | 2 | 5 | 1 | 1 | 2 |
| *M. velutinosus* CDC-B5328 | 6 | 5 | 4 | 1 | 0 | 5 | 1 | 6 | 1 | 1 | 2 |
| *M. indicus* CDC-B7402 | 7 | 8 | 4 | 3 | 0 | 8 | 1 | 4 | 1 | 3 | 3 |
