# Supplementary material for: Comparative genomics applied to Mucor species with different lifestyles
Source: BMC Genomics. 2020 Feb 10;21:135. doi: 10.1186/s12864-019-6256-2 (PMC7011435; doi:10.1186/s12864-019-6256-2)
Supplement: Supplementary file 5 — Additional file 5: Table S2. Extraction and sequencing information corresponding to the four newly sequenced Mucor isolates [file 12864_2019_6256_MOESM5_ESM.doc]

**Table S2: Extraction and sequencing information corresponding to the four newly sequenced *Mucor* isolates**

| **Isolate** | **Extraction protocol** | **Sequencing type** | **Sequencing date** | **Sequencing place** | **# million filtered reads** |
| --- | --- | --- | --- | --- | --- |
| *M. racemosus*  UBOCC-A-109155 | CATB + phenol-chloroform purifications | Paired-end  (Illumina) | 2012 | BGI | 37 |
| *M. fuscus* UBOCC-A-109160 | CATB + phenol-chloroform purifications | Paired-end  (Illumina) | 2012 | BGI | 19 |
| *M. lanceolatus*  UBOCC-A-109153 | [73] | Paired-end  (Illumina) | 2013 | Biogenouest Génomique | 74 |
|  | CATB + Qiagen | Mate-pair  (Illumina) | 2016 | Macrogen | 11 |
| *M. endophyticus*  *CBS 385-95* | CATB + Qiagen | Paired-end  (Illumina) | 2016 | Macrogen | 60 |
|  | CATB + Qiagen | Mate-pair  (Illumina) | 2016 | Macrogen | 19 |
